# LncmiRHG-MIR100HG: A new budding star in cancer

**DOI:** 10.3389/fonc.2022.997532

**Published:** 2022-09-23

**Authors:** Yingnan Wu, Zhenzhen Wang, Shan Yu, Dongzhe Liu, Litao Sun

**Affiliations:** ^1^ Cancer Center, Department of Ultrasound Medicine, Zhejiang Provincial People’s Hospital, Affiliated People’s Hospital of Hangzhou Medical College, Hangzhou, China; ^2^ Department of Pathology, The 2nd Affiliated Hospital of Harbin Medical University, Harbin, China; ^3^ Department of Hematology and Oncology, International Cancer Center, Shenzhen Key Laboratory, Shenzhen University General Hospital, Shenzhen University Clinical Medical Academy, Shenzhen University Health Science Center, Shenzhen, China

**Keywords:** lncRNA, lncmiRHG, MIR100HG, cancer, biomarker, therapeutic target

## Abstract

MIR100HG, also known as lncRNA mir-100-let-7a-2-mir-125b-1 cluster host gene, is a new and critical regulator in cancers in recent years. MIR100HG is dysregulated in various cancers and plays an oncogenic or tumor-suppressive role, which participates in many tumor cell biology processes and cancer-related pathways. The errant expression of MIR100HG has inspired people to investigate the function of MIR100HG and its diagnostic and therapeutic potential in cancers. Many studies have indicated that dysregulated expression of MIR100HG is markedly correlated with poor prognosis and clinicopathological features. In this review, we will highlight the characteristics and introduce the role of MIR100HG in different cancers, and summarize the molecular mechanism, pathways, chemoresistance, and current research progress of MIR100HG in cancers. Furthermore, some open questions in this rapidly advancing field are proposed. These updates clarify our understanding of MIR100HG in cancers, which may pave the way for the application of MIR100HG-targeting approaches in future cancer diagnosis, prognosis, and therapy.

## Introduction

Cancer is the primary barrier to increasing life expectancy in the countries of the world for its multistage development process and genetic and environmental factors (1, 2). According to the report from the international cancer research team in 2020, the number of new cancer cases and cancer-related death number increased by about 19.3 million and 10 million in the world, respectively (2). Although the understanding of molecular mechanisms in cancers has increased substantially in recent years (3, 4), the recurrence and death rates of cancer patients are still not satisfactory (2). Thus, there is an urgent need to find new effective biomarkers and treatment targets.

Long noncoding RNA (lncRNA) is over 200 nucleotides in length, with few exons, weak constraints on evolutionary processes, relatively low abundance, and lacking obvious open reading frames, resulting in protein-coding defects (5, 6). These transcripts are transcribed by RNA polymerase II and exhibit representative mRNA-like characteristics (7), and many lncRNAs are evolutionarily not conserved (8). At the molecular level, increasing evidence suggests that lncRNAs perform chromatin organization, epigenetic, transcriptional, and post-transcriptional RNA regulation through multiple mechanisms (9–13). Therefore, lncRNAs may function as tumor-suppressive and oncogenic genes, affecting the malignant behaviors of cancer cells. Moreover, the expression levels of lncRNA are dynamically modulated in a tissue, cell, or development-specific way. Consequently, lncRNA has been regarded as a promising biomarker for diagnosis and prognosis or treatment targets of tumors (14).

MiRNA-host gene lncRNAs (lnc-miRHGs) are a special type of lncRNA. Different from the various subclasses of lncRNAs that were previously categorized according to functions, genomic locations, or expression patterns (15), lncmiRHGs refer to lncRNAs containing miRNAs in exon or intron sequences (16). Typically, about 17.5% of miRNAs are derived from lncRNAs (17). At present, the biogenesis and function of lnc-miRHG-processed miRNAs are adequately studied. Some lnc-miRHGs are abnormally expressed in different disorders with promising diagnostic and prognostic biomarkers potentials. Nevertheless, it is hardly known whether an accurate and stable splice pool of lnc-miRHGs derived from the pri-miRHG during the miRNA process performs a required cellular function or no function as a by-product of miRNA processing. So far, limited research has revealed miRNA-independent functions of lnc-miRHGs, such as lncRNA H19, RMST, PVT1, and MIR31HG (18). Nevertheless, the roles of lnc-miRHGs in diverse cancers are still not studied.

LncRNA MIR100HG is one of the recently studied lncmiRHGs subclasses, encoding three miRNAs (mir-125b-1, 100, and let-7a-2) within introns. Emerging evidence showed that MIR100HG expression levels were increasing in multiple tumors in comparison to normal tissues (19–22). Besides, MIR100HG overexpression remarkably enhances tumor behaviors (23–26). However, controversial literature also reported that MIR100HG played an important role in reducing the proliferation and invasiveness of tumor cells (27, 28). However, there is still no substantial progress in the clinical application of MIR100HG.

In this review, we will highlight the characteristics, mechanisms, pathways, chemoresistance, and current research progress of MIR100HG in expression patterns, functions, and clinicopathological characteristics of cancers.

## Identification and characterization of MIR100HG

**Figure 1:**
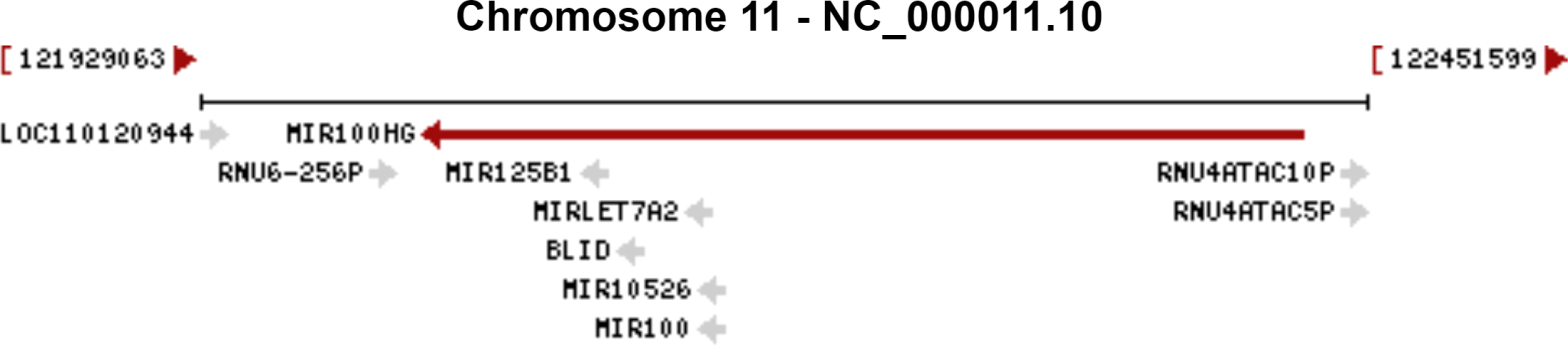
The genomic context of the MIR100HG. The genomic context of the MIR100HG was extracted from NCBI database (http://www.ncbi.nlm.nih.gov/gene/399959). Source: U.S. National Library of Medicine.

**Figure 2:**
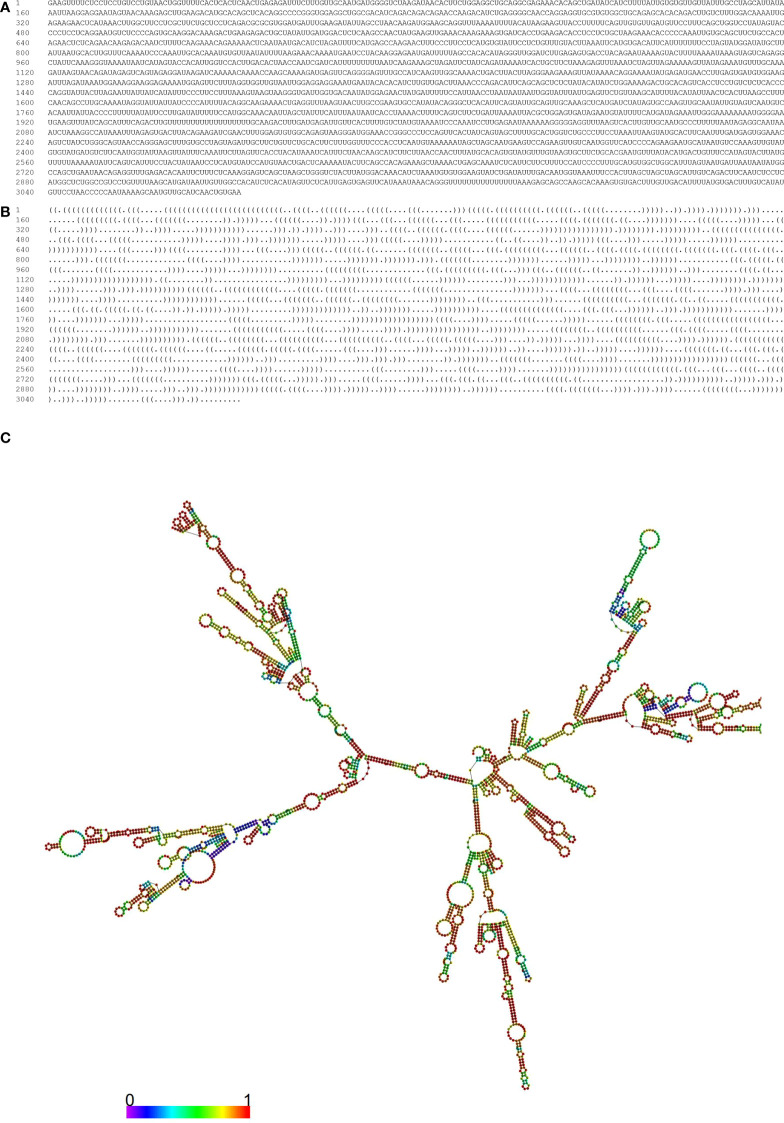
Optimal secondary structure of the MIR100HG. Prediction of the optimal secondary structure of the MIR100HG (Forena format) with-746.40 kcal/mol with its dot-bracket notation using the R-fold web server. **(A)** sequence; **(B)** dot–bracket notation; **(C)** the optimal secondary structure of the MIR100HG. In this structure, the color of the nucleotides is as follows: A, yellow; U, blue; C, green; G, red.

**Figure 3:**
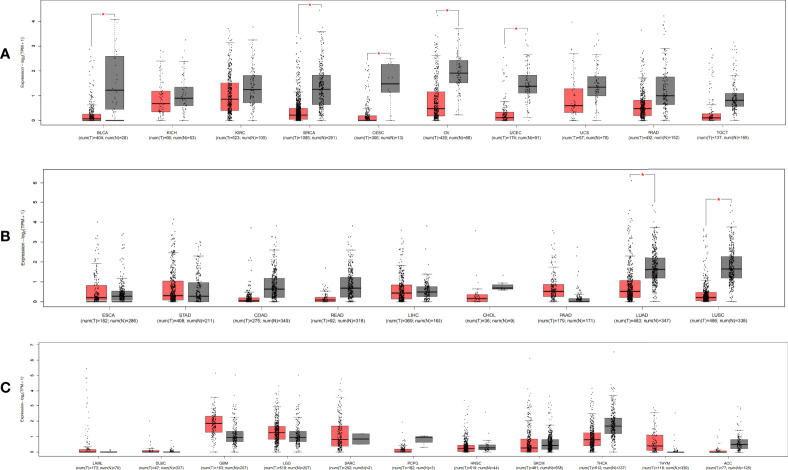
The relative expression level of MIR100HG in pan-cancer and adjacent normal tissues. The expression level of MIR100HG in different tissue of the digestive and respiratory systems. **(A)** urogenital system; **(B)** and other systems; **(C)** * |Log2FC|>1, *p* < 0.01. Data extracted from GEPIA2, all abbreviations in this figure refers to TCGA database (**Supplementary Table 1**).

**Figure 4:**
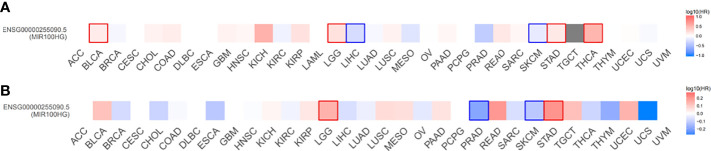
Overall survival **(A)** and disease-free survival **(B)** significance map of MIR100HG. The cut-off value was determined using the quartile of MIR100HG expression. Significance was defined as *p* < 0.05 and labeled with bold. Red represents high MIR100HG expression which suggests worse overall survival and disease-free survival; Blue represents high MIR100HG expression which suggests favorable overall survival and disease-free survival. Data extracted from GEPIA2, all abbreviations in this figure refers to TCGA database (**Supplementary Table 2**).

MIR100HG, also known as lncRNA mir-100-let-7a-2-mir-125b-1 cluster host gene, lncRNA-N2, linc-NeD125, or AGD1, is a lnc-miRHG located on chromosome 11q24.1 with 17 exons. MIR100HG has an intronic coding region (BLID), which acts as a pro-apoptotic element through the caspase-dependent mitochondrial cell death pathway (29)(**Figure 1**).103 transcripts of MIR100HG were found from the LNCipedia database with the length ranging from 242 to 7061 bp (extracted from NCBI database). MIR100HG is mostly located in the nucleus but also little in the cytoplasm (30). Non-reference sequence annotated MIR100HG is a predominantly cytoplasmic, neuronal-inducible, long intergenic noncoding RNA that includes miR-125b- 1 in introns but does not contain let-7a-2 and miR-100 and therefore renamed neuronal differentiation lncRNA host miR-125 (linc-NeD125). Linc-NeD125 not only acts as a miRNA precursor, but other features suggest that it may also have intrinsic functions, such as its specific induction of neuronal differentiation, advanced evolutionary conservation in primates, and accumulation as a spliced stable molecule under differentiation stimuli (28). While lncRNA-N2 contains the miRNAs mir-125b and let7 within its introns but does not contain miR-100. LncRNAs-N2 were expressed in brain structures. It is reported that host miRNAs are critical to neurogenesis. LncRNA-N2 promotes neurogenesis by maintaining the levels of miR-125b and let7a in neural progenitor cells. The best secondary structure prediction of MIR100HG by the R fold Web server (from the Vienna software package) has a minimum free energy of -746.40 kcal/mol, and the dotted brackets are drawn with the results illustrated in **Figure 2**. MIR100HG was originally discovered in a human transcriptome analysis (31), identified as a key role in neural stem cell neuronal differentiation (32) and mesenchymal stem cell fate determination (33), and then discovered in diverse malignant tumors (19–22). Results from the normal tissues of the Human Protein Atlas (HPA) project RNA-seq demonstrated the broad expression of MIR100HGs in the ovary (RPKM 32.2) and gall bladder (RPKM 20.3) compared with other human tissues. To explore the MIR100HG expression in multiple tumors, MIR100HG expression levels were evaluated by the GEPIA2 tool according to the Cancer Genome Atlas (TCGA) database. The results demonstrated that MIR100HG was remarkably downregulated in multiple malignant tumors of the urogenital system, including bladder urothelial cancer, ovarian serous cystadenocarcinoma, breast invasive cancer, endocervical adenocarcinoma, uterine corpus endometrial cancer, and some cancers of the respiratory system, containing lung squamous cell cancer and lung adenocarcinoma (**Figure 3**). Furthermore, the prognostic value of MIR100HG expression levels in diverse carcinomas was analyzed *via* the GEPIA2 tool. Higher MIR100HG expression suggested shorter overall survival (OS) and disease-free survival (DFS) in brain glioma with lower grade and stomach adenocarcinoma and showed favorable OS and DFS in skin cutaneous melanoma (**Figure 4**). These results revealed that MIR100HG may function as diagnostic and prognostic predictors in diverse malignant tumors.

## Expression regulation, pattern, functions, and clinicopathological characteristics of MIR100HG

**Figure 5:**
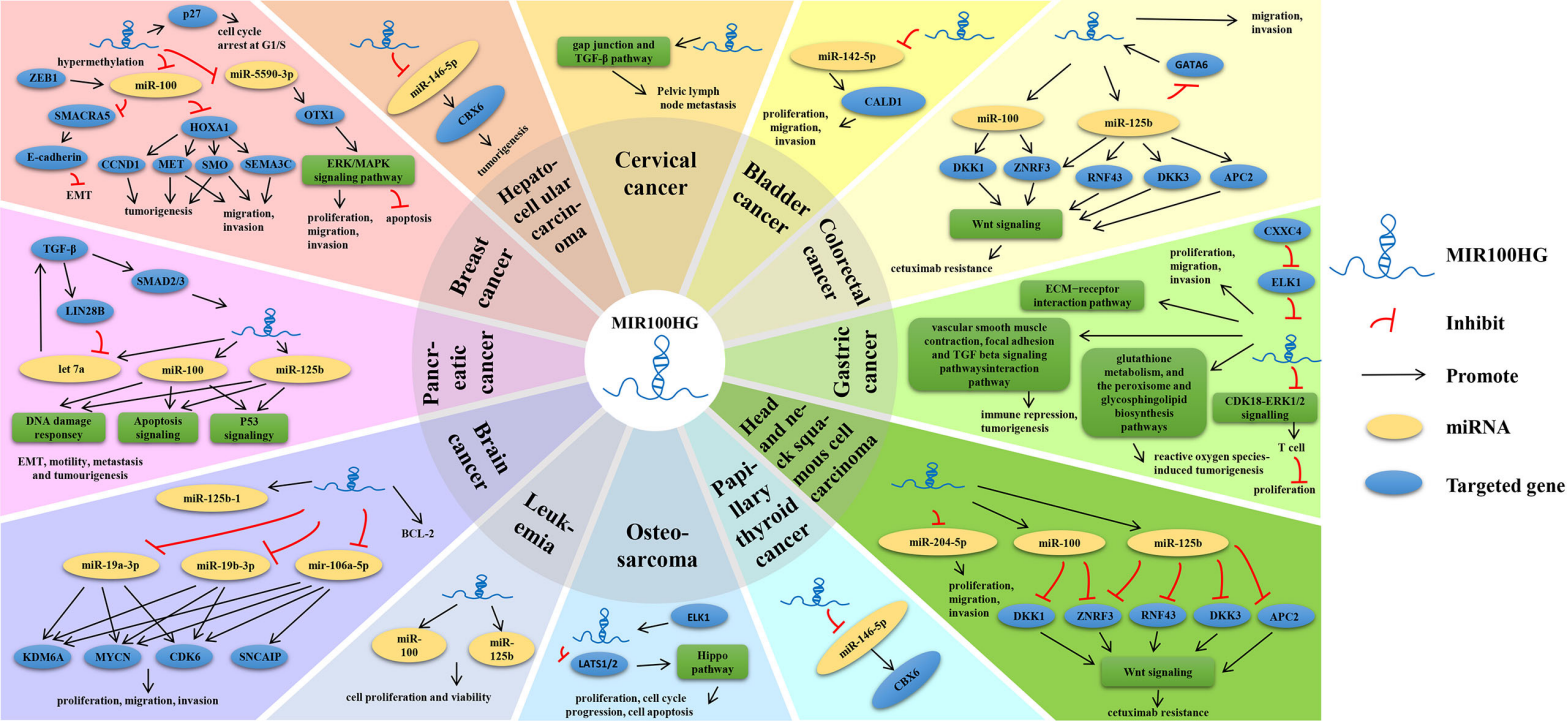
Multiple known regulatory mechanisms of MIR100HG in various human cancers.

The expression pattern, functions, and clinicopathological characteristics of MIR100HG in diverse cancers have been summarized (**Tables 1**, **2**; **Figure 5**).

**Table 1:** Functional characterization of MIR100HG in cancers.

Cancer types	Expression	Model used	Functions	Genes/proteins/pathways affected	Role	References
Brain tumor	Upregulated/Downregulated	Cell lines Human tissues	Cell apoptosis, cycle proliferation, migration, and invasion	BCL-2, miR-19a-3p, miR-19b-3p, mir-106a-5p, CDK6, MYCN, SNCAIP, and KDM6A	Oncogenic/Suppressive	(27, 28)
Head and neck squamous cell carcinoma	Upregulated	TCGA database Cell lines Human tissues	Cell proliferation, migration, invasion, and chemoresistance	Variant rs1816158, miR-204-5p, miR-100, miR-125b, DKK1, ZNRF3, RNF43, DKK3, APC2, and Wnt signal pathway	Oncogenic	(24, 34–36)
Papillary thyroid cancer	Downregulated	TCGA database	–	Hsa-miR-34a-5p and CDHR3	Suppressive	(37)
Breast cancer	Upregulated	TCGA database Cell lines Human tissues Mice	Cell apoptosis, cycle proliferation, migration, invasion, and EMT	OTX1, p27, p21, cyclin D1, miR-5590-3p, mi100, SMARCA5, E-cadherin, MET, SMO, SEMA3C, CCND1, ERK/MAPK signal pathway, and cell cycle signal pathway	Oncogenic	(19, 20, 38)
Hepatocellular carcinoma	Upregulated	Cell lines Human tissues	Cell viability, migration, and invasion	MiR-146b-5p and CBX6	Oncogenic	(39)
Lung cancer	Downregulated	Array Express database Human tissues	–	–	Suppressive	(40)
Gastric cancer	Upregulated	GEO and TCGA database Cell lines Human tissues	Cell proliferation, migration, invasion, activation of T cells, immune escape, and chemoresistance	Dilated cardiomyopathy, the glutathione metabolism, peroxisome and glycosphingolipid biosynthesis, vascular smooth muscle contraction, focal adhesion, cGMP−PKG, calcium signaling, TGF beta signaling pathways, muscle organ development, cytoskeleton organization, muscle contraction biological process CXXC4, and CDK18-ERK1/2 axis.	Oncogenic	(25, 26, 41–43)
Pancreatic ductal adenocarcinoma	Upregulated	TCGA database Cell lines Human tissues Mice	Stemness, EMT, motility, metastasis, and tumorigenesis	TGF-β, LIN28B, miR-100, miR-125b-1, let-7a, SMAD2/3/4, TP53, apoptosis, and DNA damage crucial pathways	Oncogenic	(44, 45)
Colorectal cancer	Upregulated	TCGA database Cell lines Human tissues Mice	Cell migration, invasion, and chemoresistance, EMT, and cycle arrest	HNRNPA2B1, TCF7L2, miR-100, miR-125b, Wnt signal pathway, DKK1, ZNRF3, RNF43, DKK, APC2, β-catenin, HDAC6, and p57	Oncogenic	(23, 24, 46, 47)
Bladder cancer	Upregulated/ Downregulated	Cell lines Human tissues Mice	Cell proliferation, migration, invasion, and tumor formation	HNRNPA2B1, miR-142-5p, and CALD1	Oncogenic/Suppressive	(48–52)
Cervical cancer	Downregulated	GEO and TCGA database Cell lines Human tissues	–	Gap junction and TGF-β signal pathway	Suppressive	(21, 22, 53, 54)
Osteosarcoma	Upregulated	GWA data Cell lines Human tissues Dogs	Cell proliferation, cycle arrest, and apoptosis	ELK1, EZH2, LATS1/2, Hippo, PI3K and Rb signal pathway	Oncogenic	(55, 56)
Leukemia	Upregulated	Cell lines Human tissues	Cell proliferation, viability, apoptosis, and necrosis	MiR-100, miR-125b-1, and TGFβ	Oncogenic	(57–60)

**Table 2:** Clinical significance of MIR100HG in cancers.

Cancer types	Model used	Clinicopathologic features	References
Head and neck squamous cell carcinoma	TCGA database Human tissues	AJCC stage, resistance, and overall survival	(24, 34, 36)
Papillary thyroid cancer	GEO and TCGA database	Overall survival	(61)
Breast cancer	Mice	Larger tumor size and poor survival	(19, 20)
Hepatocellular carcinoma	**Human tissues**	TNM stage and Edmondson-Steiner grading	(39)
Gastric cancer	GEO and TCGA database Human tissues	Survival time, TNM stage, clinical stage, tumor invasion, lymph node metastasis, distant metastasis, overall survival, and disease-free survival	(25, 41, 42)
Pancreatic ductal adenocarcinoma	Human tissues	Overall survival and disease-free survival	(44)
Colorectal cancer	TCGA database Human tissues	T stage, lymph node metastasis, distant metastasis, AJCC stage, histological differentiation, poor survival, and unfavorable prognosis	(23, 46)
Bladder cancer	GEO and TCGA database Human tissues	Poor clinical outcome, poor prognosis, chemosensitivity, histological grade and clinical stage	(48, 50, 51)
Cervical cancer	GEO and TCGA database Human tissues	Lymph node metastasis and poor prognosis	(21, 53)
Osteosarcoma	Human tissues	Tumor size, advanced clinical stage, and poor prognosis	(55)

### Brain tumors

Medulloblastoma (MB) is the most common malignant brain tumor in children (62). Among them, group 4 medulloblastoma (G4MB) and type 3 medulloblastoma (G3MB) account for about 60%, and the prognosis is relatively poor, especially since the targeted therapy of G3MB is still in its infancy, and the pathogenesis is unclear (63). Laneve et al. (28) found that the expression of linc-NeD125 was significantly higher in G4MB than in normal brain tissues, and its expression was also significantly higher than the other three types of MBs. While Kesherwan et al (64) found that MIR100HG was significantly down-regulated in G3MB. Downregulation of linc-NeD125 reduces G4 cell proliferation. In addition, G3MB has a worse prognosis than G4MB. Interestingly, overexpression of linc-NeD125 in the G3MB cell model acquired specific G4MB molecular signatures. In other words, overexpression of linc-NeD125 in the G3MB cell model increases the protein production of the G4MB driver gene with a significant reduction of the high proliferation, migration, and invasion of G3MB cells, which gives them capabilities that G4MB-like cells possess. Therefore, the action mode of linc-NeD125 in MB deserves a more in-depth study for possible therapeutic applications. Controversially, linc-NeD125 and its contained miR-125b-1 function as negative regulators in human neuroblastoma cell proliferation, apoptosis, and viability. Linc-NeD125 can reduce cell proliferation and activate the anti-apoptotic factor BCL-2, and mainly controls cell viability by preventing cell cycle progression independently of host miRNAs (27). Therefore, linc-NeD125 may play different regulatory roles in different brain tumors, and the related mechanisms need to be further studied.

### Head and neck squamous cell carcinoma

Most squamous cell carcinomas of the head and neck are diagnosed as advanced, which is the sixth most common carcinoma in the world (65, 66). Wilkins et al. (34) performed a genome-scale assessment of the OS contribution of common RNA SNPs to HNSCC and site-specific disease and found that the MIR100HG variant rs1816158 is related to OS in oral cancer. Similarly, Zhou et al. (35) also discovered that MIR100HG was significantly associated with OS in patients with tongue squamous cell carcinoma. Meanwhile, expression of MIR100HG-derived miR-100 was significantly related to OS in head and neck cancers, while other MIR100HG-derived miRs, 125b-1 and let7a-2, were not related to OS. The C allele in rs1816158, which is related to an increased risk of death, increases miR-100 expression. Another study discovered that the MIR100HG was up-regulated in the tumor tissue of laryngeal squamous cell carcinoma (LSCC) patients, while miR-204-5p was down-regulated. And their significant negative correlation was not identified in adjacent healthy tissues, indicating that there is a pathological mediator between the two RNAs. MIR100HG may promote the proliferation, migration, and invasion of cancer cells in LSCC *via* downregulating miR-204-5p, while overexpression of miR-204-5p merely partially reduced the enhancement of MIR100HG overexpression on the malignant behaviors of cancer cells, indicating MIR100HG may also have multiple downstream effectors in LSCC. But the pathological mediators and downstream effectors haven’t been revealed. Besides, the expression of MIR100HG was significantly increased with the increase of AJCC staging, while the expression of miR-204-5p was significantly decreased (36). Moreover, MIR100HG, miR-100, and miR125b were simultaneously upregulated in HNSCC cell lines which were acquired and resisted in new cetuximab, while inhibition of MIR100HG, miR-100, and miR125b can restore cetuximab responsiveness *in vitro* and vivo, which also provides novel ideas for the therapeutic methods of HNSCC cetuximab resistance (24). These results demonstrated that MIR100HG acts as an oncogenic lncRNA in head and neck cancers, which may represent a potential diagnostic biomarker or a novel therapeutic target for cancers.

### Papillary thyroid cancer

Papillary thyroid cancer (PTC) is the most common subtype of thyroid cancer. Yang et al. (37)identified that MIR100HG was differentially expressed between cancerous and normal tissues and significantly downregulated in PTC tumors. Additionally, MIR100HG was co-expressed with CDHR3, which was targeted by hsa-miR-34a-5p. The epigenetic regulation among hsa-miR-34a-5p, CDHR3, and MIR100HG may participate in the pathological mechanism of PTC. MIR100HG was significantly but not independently associated with OS of PTC patients (61). These results indicated that MIR100HG may promote the progress of PTC by sponging the hsa-miR-34a-5p/CDHR3 axis, providing a potential marker in PTC patients. However, the expression and regulation mechanism between them has not been validated.

### Breast cancer

Breast cancer is the most common malignant tumor in women and has become the main cause of cancer-related death. It can be divided into different subtypes. Triple-negative breast cancer (TNBC) is the most aggressive and high-risk occurrence subtype (67, 68). Chen et al. (38) showed that MIR100HG was significantly up-regulated in TNBC tissues and cells, and knockdown of MIR100HG inhibited the growth of TNBC cells and tumors *in vivo* and vitro. Consistently, Wang et al. (19) discovered that MIR100HG can promote the proliferation of TNBC cells. Overexpression of MIR100HG in TNBC MDA-MB-231 cells could increase the proportion of cells in the S phase, while knockdown of MIR100HG resulted in cell arrest in the G1 phase. Meanwhile, MIR100HG could promote DNA replication in TNBC cells. However, the coding potential calculator used to predict translational capacity showed that MIR100HG had no potential or ability to convert to protein. The expression of MIR100HG was significantly higher in TNBC than in other subtypes based on TCGA database. Wang et al. (20) further verified at the cellular level and found that MIR100HG could be detected in a variety of breast cancer cell lines, and its expression level in TNBC cell lines was indeed higher than in others. High expression of MIR100HG was related to a low survival rate. Additionally, Chen et al. (38) found that MIR100HG could also augment the expression of the OTX1 gene *via* sponging the miR-5590-3p, thereby promoting the malignant behaviors of TNBC cells, and inhibiting the apoptosis and cell cycle arrest in G0/G1 phase. Mir-100 embedded in the host gene MIR100HG also suppressed tumorigenicity, motility, and invasiveness of breast tumor cells besides miR-5590-3p, but miR-100 was generally downregulated in all subtypes. DNA hypermethylation of MIR100HG may lead to the downregulation or loss of the miR-100, while miR-100 expression is not only regulated by the aforementioned epigenetic silencing but also regulated by transcriptional activation. ZEB1 is usually observed in triple-negative and basal-like breast tumors and the activity of the mir-100 promoter is also significantly increased. Although it remains controversial whether EMT is related to increased tumorigenicity (38). Chen et al. (38) found that miR-100 is a new EMT inducer. EMT induction and tumor inhibition of mir-100 are conducted at different targets. Specifically, miR-100 inhibits EMT by targeting the CDH1 promoter methylation regulator SMARCA5 to downregulate E-cadherin. Moreover, miR-100 can also inhibit its multiple downstream targets involved in tumorigenesis by directly targeting HOXA1, such as MET, SMO, and CCND1, and downstream targets SEMA3C, MET, and SMO involved in cell migration and invasion. Generally, it is believed that MIR100HG may be an oncogene in TNBC and may serve as a potential prognostic biomarker and therapeutic target for TNBC. Currently, there is no clear treatment guideline for TNBC. Therefore, it is necessary to further study the regulatory mechanism of MIR100HG in TNBC.

### Lung cancer

Lung cancer is the second most common cancer in the world in 2020 and the main cause of cancer-related death (69). Non-small-cell lung cancer (NSCLC) accounts for approximately 80%-85% of all subtypes. Yu et al. (40) performed differential expression analysis on the GSE19188 microarray dataset obtained from the ArrayExpress database, including 91 tumor tissues and 65 adjacent non-cancer tissues, and found that the expression of MIR100HG was reduced in tumor tissue compared with non-cancer tissue samples, but there was no differential expression between histological categories. Moreover, the HG-U133Plus2.0 array used by the researchers can only represent some but not all of the potential lncRNAs. As mentioned above, MIR100HG may represent a potential therapeutic target for NSCLC patients. However, there is still limited information on the possible biological functions and mechanisms of MIR100HG. Thus, more comprehensive and in-depth analytical studies are urgently required to clarify the function of MIR100HG in lung cancer.

### Hepatocellular carcinoma

Hepatocellular carcinoma (HCC) is common primary liver cancer, which is accompanied by chronic viral hepatitis B or C and cirrhosis. Li et al. (39) discovered that MIR100HG expression was upregulated in HCC patient tissues and cells and related to the HCC progression. Increased MIR100HG expression was related to advanced TNM stage (III+IV) and Edmondson-Steiner grading (III+IV) in HCC patients and can promote the viability, migration, and invasion of HCC Cells. Bioinformatics analysis results demonstrated that miR-146b-5p was directly targeted by MIR100HG, while the binding site of miR-146b-5p was located on the 3′UTR of CBX6. The elimination of MiR-146b-5p or the overexpression of CBX6 can inhibit the MIR100HG suppression on tumorigenesis of HCC cells (39). However, the specific functional pathway has not been revealed. In summary, MIR100HG may represent a new biomarker for HCC.

### Gastric cancer

Gastric cancer (GC) is the fourth most common tumor type and the second most common cancer-related death in the world (70). It is found that MIR100HG was highly expressed in gastric cancer by bioinformatics methods, and its expression was significantly related to the TNM stage, but not with lymph node metastasis (LNM) or age (25). Differently, another research group (41) further explored and declared that high expression of MIR100HG has a positive correlation with clinical stage, invasion, and lymph node and distant metastasis, but not with gender, age, histological grade, and HP infection. Furthermore, high expression of lncRNA MIR100HG predicts short OS in GC patients, and high MIR100HG expression is also an independent adverse prognostic factor of OS in GC patients. Additionally, Wu et al. (42) identified lncRNA MIR100HG, LINC00205, TRHDE-AS1, OVAAL, and LINC00106 as independent prognostic factors of GC from TCGA and GEO databases by bioinformatics analysis. LINC00106 was considered to be a protective factor. The remaining four lncRNAs are risk factors. They established a risk score model including these five lncRNAs to predict OS and DFS in GC patients, especially in stage II-IV GC patients. Another group (43) also constructed a lncRNA signature including MIR100HG and other 10 lncRNAs, which was associated with the prognosis of GC and can be a more effective prognostic factor for GC patients.

Further experiments *in vitro* showed that low expression of MIR100HG inhibited proliferation, migration, and invasion of GC cells. Recently, a research group (26) analyzed the GC dataset through the GEO database and revealed that CXXC4 was a significantly down-regulated gene in GC. In contrast, ELK1 was significantly upregulated in GC. CXXC4 and ELK1 were identified to be co-expressed in GCs. ELK1 and MIR100HG were also co-expressed. Simultaneously, JASPAR prediction showed that ELK1 binds to the MIR100HG promoter region. Overexpression of CXXC4 can inhibit the phosphorylation of ELK1 and reduce its nuclear translocation, resulting in weakened binding of ELK1 to the MIR100HG promoter, which in turn inhibits CDK18-ERK1/2 signaling, promotes T cell activation, and activates CD3+ T cells. The increased secretion of IFN-γ inhibited the immune escape of GC cells and decreased the proliferative capacity of GC cells. Inhibition of the CXXC4/ELK1/MIR100HG pathway suppressed immune evasion of GC cells, which highlights a possible treatment target for GC. Overall, MIR100HG may serve as a novel target for the diagnosis and treatment of GC.

### Pancreatic ductal adenocarcinoma

Pancreatic duct adenocarcinoma (PDAC) is a high mortality cancer with about a 6% survival rate of 5 years (71). Ottaviani et al. (44, 45) revealed that TGF-β can directly promote the transcription of MIR100HG by activating SMAD2/3/4 transcription factors (TFs). Although MIR100HG derived miR-100, let-7a, and miR125b, only miR-100 and miR-125b were up-regulated, and let-7a was unchanged, thereby promoting epithelial-to-mesenchymal transition (EMT) and tumorigenesis in PDAC cells. miR-125b is the most important effector of TGF-β-mediated tumorigenesis, as inhibition of miR-125b or miR-100 affects the ability of TGF-β to induce cell motility and promote spindle cells, but only miR-125b was able to reverse the ability of TGF-β to induce tumorigenesis *in vivo* and vitro. Phenotypically, miR-100 and miR-125b are involved in the EMT, motility, metastasis, stemness formation, and tumorigenesis of PDAC. Moreover, high levels of the two miRNAs were associated with decreased OS and DFS in PDAC patients. They can regulate key pathways in PDAC progressions, such as apoptosis, TP53, and DNA damage, and also regulate many genes involved in suppressing p53 and DNA damage response pathways important for this common metastatic disease progression. However, more efforts should be devoted to illustrating other regulatory mechanisms and clinical implications of MIR100HG in PADC. Of note, MIR100HG may become a promising candidate for PDAC targeted therapy.

### Colorectal cancer

Colorectal cancer (CRC) has a high mortality rate, and tumor invasion and distant metastasis are the main causes of cancer-related death in CRC patients (72). MIR100HG, miR-100, and miR-125b may be potential predictive biomarkers for cetuximab (CTX) resistance. CRC cell lines were accompanied by overexpression of MIR100HG, mir-100, and mir-125b in the case of new and acquired CTX resistance. Similarly, this may also occur in patients with KRAS/NRAS/BRAF mutations and CTX resistance. Besides, analysis of the TCGA CRC database demonstrated a stage-dependent increase in the expression of MIR100HG (24). In further experiments, Li et al. (23) found that MIR100HG was significantly increased in CRC tissues compared with normal mucosal tissues. The expression of MIR100HG in advanced stage tissues was significantly higher than that in the early stage and may be related to the aggressive phenotype of CRC patients. Furthermore, MIR100HG overexpression may be an important factor in CRC metastasis, prognosis, and survival. The high expression of MIR100HG showed a positive correlation with TNM stage, lymph node and distant metastasis, AJCC stage, and tumor histological differentiation. However, there was no correlation between high MIR100HG expression and clinical parameters, such as age, sex, tumor size, tumor location, and vascular invasion status. The DFS and OS of CRC patients with high MIR100HG expression were shorter than those of patients with low MIR100HG expression. In addition, MIR100HG expression, AJCC stage, T classification, N classification, M classification, and tumor differentiation could be considered independent prognostic factors for DFS and OS. The expression of MIR100HG was related to EMT markers and was a positive regulator of EMT. Different experiments *in vitro* and vivo still confirmed that MIR100HG sustained cetuximab resistance and overexpression of MIR100HG promoted migration and invasion of CRC cells *in vitro*, as well as the liver metastasis ability *in vivo (* 46). Overexpression of MIR100HG induced cell cycle G0/G1 arrest and repressed cell proliferation *via* p57 upregulation *in vitro* and *in vivo (* 47). Above all, MIR100HG plays a key role in CRC progression and serves as a novel prognostic biomarker for CRC. The regulation of MIR100HG expression level may be a potential treatment strategy for CRC patients, especially those with liver metastases. Although MIR100HG acts as a miRNA host gene, its overexpression can upregulate miR-100 and miR-125b, there are few studies on the effect of MIR100HG on the expression levels of miR-100 and miR-125b in CRC. Therefore, the effect of regulating MIR100HG expression on the expression levels of miR-100 and -125b, and how MIR100HG promotes the progression of CRC remains to be studied. Overall, it is believed that MIR100HG may serve as a potential prognostic biomarker and therapeutic target for CRC.

### Bladder cancer

Bladder cancer (BC) is the second most common cancer in men worldwide. It has multiple genetic as well as phenotypic features. Even with systemic therapy, most patients eventually relapse or metastasize (73). Muscle-invasive bladder cancer (MIBC) is considered to be the main cause of BC-related death (74). Nevertheless, its mechanism remains largely unknown. Wang et al. (48) conducted a comprehensive analysis of mRNA, lncRNAs, and miRNAs between MIBC and non-tumor bladder samples based on the TCGA database and found that MIR100HG was significantly differentially expressed between MIBC and normal samples and was downregulated in MIBC. On the contrary, Zhang et al. (49) found that MIR100HG was significantly up-regulated in BC tissues in comparison to adjacent tissues, and its high expression was positively correlated with the histological grade and clinical stage of BC. Besides, the expression of MIR100HG was negatively related to the OS of patients (50). MIR100HG is an independent prognostic factor for BC. Additionally, the genomic instability-related lncRNA signature including MIR100HG and other four lncRNAs (CFAP58-DT, LINC02446, AC078880.3, and LINC01833) was also an independent prognostic factor of BC (51). In further experimental studies, Ying et al. (52) discovered that high expression of MIR100HG inhibits the proliferation and invasion of BC cells, and HNRNPA2B1 expression is down-regulated while MIR100HG is overexpressed. Additionally, overexpression of MIR100HG inhibits tumor formation in nude mice *in vivo*. However, Zhang et al. (49) found that high expression of MIR100HG significantly enhanced the proliferation, migration, and invasion abilities of BC cells. Taken together, these findings suggested that MIR100HG may play a vital role in BC and more studies should be replicated for the controversial results to validate the specificity and sensitivity of the MIR100HG product or individual MIR100HG as biomarkers.

### Cervical cancer

Carcinoma of the uterine cervix (CACX) is the fourth leading cause of female cancer death (2). Papillomavirus infection is a major causative factor for CACX in high-risk populations, and the long latency period for tumor development after the infection has other genetic and epigenetic changes that lead to deregulation of cellular pathways, which in turn affect protein-coding genes and non-protein-coding genes (75). MIR100HG was found significantly differentially expressed and down-regulated in CACX (53) and CACX patients with pelvic lymph node metastasis (PLNM) (21), and pathway analysis showed a significant correlation with the “cell growth and proliferation” and “cancer” phenotypes (54). Zhang et al. (53) established a prognostic model of CACX with genomic instability-associated lncRNAs including MIR100HG, AC107464.2, and AP001527.2. Besides, the biological function of MIR100HG was correlated with promoter methylation of CACX. However, contrary to the expression result, CACX and CACX patients with PLNM with high expression of MIR100HG showed poor prognosis by Kaplan-Meier analysis based on the TCGA cohort (21, 54). PLNM is the most important prognostic parameter in CACX, especially in the early stage of CACX. Thus, effective PLNM detection is crucial for choosing the best treatment plan. GSEA showed that the RICKMAN_METASTASIS_UP gene set had a higher normal enrichment score and a positive correlation with the gene profile. Finally, this result confirms an important value of MIR100HG in the tumor LNM, which will guide further related *in vivo* and *in vitro* experimental studies and CACX metastasis treatment in the future.

### Osteosarcoma

Osteosarcoma is a typical malignant bone tumor mostly discovered in adolescents (76), and its poor prognosis is mainly due to rapid growth and early metastasis (77). MIR100HG has been revealed overexpressed in osteosarcoma cell lines and patient samples. Overexpression of MIR100HG indicated larger tumors and advanced clinical stage. In addition, the expression of MIR100HG showed a negative correlation with the OS rate, and the overexpression of MIR100HG suggested a poor prognosis. Cell function experiments showed that knockdown of MIR100HG inhibited OS cell proliferation, promoted cell apoptosis, and induced cell cycle arrest in G0/G1 phase (55). Su et al. (55) studied the upstream mechanism of MIR100HG and found that ELK1 is a potential transcriptional activator of MIR100HG. Experimental verification indicated that the 1332bp to 1323bp sites in the MIR100HG promoter region were responsible for ELK1-induced transcriptional activation. ELK1 has a high expression in both osteosarcoma tissues and cell lines and was positively associated with the expression of MIR100HG. ELK1 induces MIR100HG epigenetic silencing of LATS1/2 upregulation by binding to the histone methylation regulator EZH2. The expressions of LATS1 and LATS2 were negatively correlated with MIR100HG, and LATS1/2 could partially reduce MIR100HG-regulated cell proliferation, cell cycle progression, and apoptosis. In the same year, Zapata et al. (56) also found that MIR100HG serves as a new candidate gene that can function alone or in combination with other selected genes such as PHLPP1 and BRIP3 that are also in related PI3K and Rb pathways. Importantly, this model will shed light on cancer regulatory mechanisms and the resulting patterns of somatic mutation. Treatments validated in model organisms could enable small, gene-targeted clinical trials in pet dogs, which not only greatly improves the success rate of human trials, but also provides key information on the mechanisms of drug resistance. The above findings supported the fact that MIR100HG, as an oncogenic lncRNA, could be a novel biomarker in Osteosarcoma.

### Acute myeloid leukemia

Acute myeloid leukemia (AML) is a highly aggressive hematological tumor that usually occurs in adults. Particularly, children with Down syndrome (DS) have a 400-fold increased risk of developing acute megakaryoblastic leukemia (AMKL) (57), but DS-AMKL patients have a favorable prognosis compared with non-DS-AMKL patients. Emmrich et al. (57) found that miR-125b-2 was highly expressed in DS-AMKL and non-DS-AMKL. Whereas the polycistronic homolog of miR-99a-125b-2 on hsa21 and miR-100-125b-1 in the MIR100HG intron on hsa11 have the same configuration. MIR00HG is more expressed in erythrocytes, hematopoietic stem/progenitor cells, and B cells in comparison to other blood cell lines. The MIR100HG expression in the AMKL cell line is higher than that in other leukemia cell lines. Regression analysis confirmed that MIR100HG was positively correlated with its miRNA polycistronic. Knockdown of MIR100HG impairs cell replication efficiency and viability in AMKL cells while altering the expression of lineage surface markers, increasing the percentage of CD36+ cells and decreasing the proportion of CD41+ cells. MIR100HG induces apoptosis of human megakaryocytic leukemia cells through up-regulating the expression of TGF-β, inhibiting the proliferation of human AMKL cell lines, and inducing their apoptosis and necrosis (58, 59). MIR100HG is mainly located in the nucleus, which makes it a role in regulating erythroid megakaryocyte development, while further studies are needed to determine the protein interaction target and to pinpoint the accurate subnuclear region and DNA target sequence of MIR100HG. The above results suggest that MIR100HG may be a new marker for improving AML treatment.

Studies on acute promyelocytic leukemia (APL) have reported that the up-regulation of MIR100HG is related to the proliferation of primary APL cells. Degradation of MIR100HG by antisense LNA GapmeRs can induce apoptosis, necrosis, and inhibit cell proliferation in APL cells. Therefore, inhibition of MIR100HG is a novel approach to control APL cell proliferation, which can be considered for the treatment of APL alone or in combination with existing treatments, or for patients who are resistant to conventional APL therapy (60). Corresponding clinical trials are still necessary to verify the effectiveness of MIR100HG for APL.

## Mechanism of MIR100HG

The regulatory mechanisms of MIR100HG are diverse. According to current research, MIR100HG is mainly involved in pre-transcriptional regulation and post-transcriptional regulation. Pre-transcriptional regulation is mainly (1) as a promoter of RNA binding proteins (RNA binding proteins, RBPs) (2); as a structural component to form nucleic acid-protein complexes with proteins; post-transcriptional regulation is mainly (3) as ceRNA; (4) as miRNA precursor (**Table 1** and **Figure 5**).

### A promoter of RNA binding proteins

MIR100HG positively regulates the association between RBP HuR and multiple target mRNAs. The 3’ end of MIR100HG is rich in U sequences. MIR100HG takes advantage of the U-rich motif to interact with HuR and HuR target mRNAs (18). Consistent with the aforementioned function of MIR100HG as an RNA-binding protein promoter, Panagiotis Papoutsoglou et al. (78) extended this model in the context of TGFβ tumor biology. The 3′ untranslated region of TGFB1 mRNA contains AU-rich sequences recognized by HuR, and MIR100HG promotes the formation of a complex between the RNA-binding protein HuR and TGFβ1 mRNA, thereby stabilizing the mRNA and enhancing autocrine TGFβ1 production and autoreactivity. Researchers suggested that HuR contains three RNA recognition motifs, one of which promotes HuR dimerization. Thus, there may be a mechanism whereby MIR100HG binds to one RNA recognition motif to promote TGFβ1 mRNA and the second RNA recognition motif in the dimerized HuR sequential combination. On the other hand, this lncRNA is in turn directly regulated by RBP. The RBP TDP-43 can directly regulate the expression and control stability of the mature form of linc-NeD125 merely under differentiation conditions, while this lncRNA is stabilized (27).

### As a structural component to form nucleic acid-protein complexes with proteins

Chen et al. (38) discovered that the expression of MIR100HG was significantly up-regulated in TNBC tissues and cells and MIR100HG can affect the progression of TNBC by regulating the expression of p27. Through bioinformatics prediction, three ncRNA binding motif triplex-forming oligonucleotides (TFOs) were found in MIR100HG, which specifically bind to p27 to form an RNA-DNA triple structure. They are located at 275-352nt (TFO1), 462-557nt (TFO2), and 2635-2688nt (TFO3) in the MIR100HG sequence, respectively. But the three TFOs do not act synergistically, and TFO1 is a functional binding site on the p27 site. Generally, lncRNAs in tumors interact with the EZH2 gene by trimethylation of lysine 27 of histone 3 on the p27 promoter, inhibit p27, or directly bind and activate the p27 promoter (79, 80). MIR100HG binds to the p27 gene locus through the TFO1 sequence to form an RNA-DNA triple structure, recruits epigenetic modification proteins to directly bind and activate the p27 promoter to regulate p27 expression, and is involved in the regulation of cell proliferation in TNBC. Additionally, both p21 and p27 are cyclin-dependent kinases (CDK) inhibitors, while cyclin D1 is necessary to G1 cell cycle progression. After MIR100HG overexpression, the expression of both p21 and p27 is reduced, and cyclin D1 expression is increased. p21, p27, and cyclin D1 are G1/S checkpoint cell cycle regulator proteins. p21 and p27 function on the CDK4/6-cyclin D1 complex, thereby preventing cell cycle progression in the G1 phase, while cell cycle D1 facilitates the G1/S transition. However, the locus-specific formation of RNA-DNA triplet structures by MIR100HG with p21 or cyclin D1 has not been discovered in bioinformatics predictions, but it cannot be ruled out that MIR100HG may also directly regulate p21 and cyclin D1 through other mechanisms. But it can’t be ruled out that MIR100HG is possible to regulate p21 and cyclin D1 through other mechanisms (20).

### As ceRNA

LncRNAs are often reported as molecular sponges that bind to miRNAs to inhibit the binding of miRNAs to target mRNAs and are involved in regulating cancer progression. However, MIR100HG does not play a role in TNBC cells through its host miRNAs but plays a critical role in the occurrence and progression of TNBC through other miRNAs in the ceRNA regulation mode. Chen et al. (38) found that MIR100HG can up-regulate the expression of the OTX1 gene by targeting miR-5590-3p, thereby promoting the malignant behaviors of TNBC cells, and inhibiting their apoptosis and cell cycle arrest in G0/G1 phase.Linc-NeD125 as a ceRNA can recruit the miRNA-induced silencing complex miRISC and directly bind the miRNAs miR-19a-3p, miR-19b-3p, and miR-106a-5p, sequestering the three miRNAs, resulting in the dysregulation of four target genes CDK6, MYCN, SNCAIP, and KDM6A, which are major drivers of G4MB (28). MIR100HG may promote the proliferation, migration, and invasion of cancer cells in LSCC by downregulating miR-204-5p, and MIR100HG may also have multiple downstream effectors in LSCC (36). MIR100HG deficiency inhibited the tumorigenesis in HCC cells by targeting the miR-146b-5p/CBX6 axis (39). MIR100HG can regulate CALD1 expression by sponging miR-142-5p to inhibit the proliferation, migration, and invasion of bladder cancer cells (49).

### As miRNA precursor

Bevilacqua et al. (27) suggested that linc-NeD125 and its miR-125b-1 function as negative regulators in human neuroblastoma cell proliferation. These two-overlapping noncoding RNAs are coordinated *in vitro* during neuronal differentiation, and their expression is regulated by distinct mechanisms. While the production of miR-125b-1 is dependent on transcriptional regulation, linc-NeD125 keeps it under control at the post-transcriptional level by regulating its stability. On the other hand, linc-NeD125 acts independently of host miRNAs by attenuating cell proliferation and activating the anti-apoptotic factor BCL-2.

## MIR100HG participates in multiple signaling pathways

The MIR100HG participates in multiple signaling pathways in diverse cancers (**Table 1**).

### Wnt/β-catenin signaling pathway

The Wnt/β-catenin signaling pathway appears repeatedly in the occurrence and progression of different cancers and is very important for regulating cell proliferation, survival, migration, and other processes. There are many inhibitors of this pathway, including the DKK family, APC, ZNRF3, RNF43, etc. (24) Lu et al. (24) discovered that MIR100HG, miR-100, and miR125b were simultaneously up-regulated under acquired and new cetuximab resistance in HNSCC and CRC cell lines. Five negative regulators (DKK1, DKK3, ZNRF3, RNF43, and APC2) of the typical Wnt signaling pathway are synergistically downregulated by miR-100 and miR-125b. DKK1 and ZNRF3 are targeted by miR-100, and ZNRF3, RNF43, DKK3, and APC2 are targeted by miR-125b to increase the Wnt/β-catenin signaling pathway, resulting in resistance to cetuximab. Inhibition of Wnt signaling restored cetuximab responsiveness *in vitro* and *in vivo*. However, effects of the full-length 3-kb MIR100HG transcript on Wnt signaling cannot be ruled out. In addition, according to the analysis of the TCGA database, the research group also found that the expression of GATA6 was decreased in stage IV CRC, while the expression of MIR100HG was increased, and the increase in the expression of miR-125b could enhance the inhibitory effect of GATA6, which in turn promoted the increased expression of MIR100HG. In this case, GATA6 can indirectly prevent cetuximab resistance caused by enhanced Wnt signaling to exert a tumor suppressor effect, which is different from previous studies in that GATA6 promotes tumorigenesis by activating Wnt signaling (81). Additionally, the study group further demonstrated that MIR100HG interacted with hnRNPA2B1 to augment Wnt signaling by stabilizing TCF7L2 mRNA, which is a core component that binds to nuclear β-catenin to promote downstream Wnt target gene transcription. hnRNPA2B1 recognized the N6-methyladenosine ­(m6A) site of TCF7L2 mRNA in the presence of MIR100HG. TCF7L2 activated MIR100HG transcription. Above all, MIR100HG functions in concert with their encoded miR-100/125b to enhance Wnt signaling activity in the setting of advanced CRC, but at different levels of Wnt signaling *via* complementary mechanisms (46). β-catenin/TCF4 can bind to the MIR100HG promoter. Additionally, MIR100HG was negatively correlated with HDAC6 and β-catenin in CRC specimens. β-catenin forced expression reduced primary and mature lnc-MIR100HG levels and the enrichment of H3K27Ac. HDAC6 was recruited to the MIR100HG promoter and downregulated H3K27Ac enrichment in a β-catenin-dependent manner (47). Above all, it provides new ideas for further therapeutic approaches.

### ERK/MAPK signaling pathway

The ERK/MAPK signaling pathway is the most studied MAPK signaling pathway (38). Chen et al. (38) found that knockdown of MIR100HG *in vitro* and *in vivo* decreased the expression of OTX1 by upregulating miR-5590-3p and successfully inhibits the activation of the ERK/MAPK signaling pathway in TNBC, thereby inhibiting tumorigenesis.

### TGF-β signaling pathway

TGFβ signaling pathway is signaling through membrane receptors. Membrane receptor activates effector transcription factors SMADs and MAPKs, which regulate target genes that control cell cycle, migration, extracellular matrix remodeling, and EMT. TGFβ can promote the stemness, invasiveness, and metastasis of advanced tumors (78). Shang et al. (21) found that MIR100HG has a key value in CACX LNM by regulating the TGF-β pathway. Moreover, Noordhuis et al. (22) confirmed that disrupting the TGF-β pathway may promote the malignant progression of cervical dysplasia in human CACX, further suggesting that MIR100HG is possible to participate in the regulation of early CACX LNM through the TGF-β pathway. Ottaviani et al. (44, 45) found that TGFβ induced MIR100HG transcription in pancreatic cancer cells. Although MIR100HG derived miR-100, let-7a, and 125b, it only induced the expression of miR125b and miR-100, but not miR let-7a -2 expression. The lack of change in let-7a may be due to the post-transcriptional downregulation of let-7a levels induced by TGF-β in LIN28B. Such a system of primary polycistronic miRNA transcript upregulation and post-transcriptional repression of specific miRNAs is critical to TGF-β responses in PDAC. In addition, various MIR100HG miRNAs mediated by TGFβ in keratinocytes and different cancer cells may reflect different physiological outcomes. TGFβ induces antitumor let-7a-2-3p to promote its antiproliferative capacity. Conversely, TGFβ often loses its antiproliferative capacity and instead promotes EMT and stem cells, including increased expression of EMT-promoting miR-100 and miR-125b in pancreatic cancer. As previously stated, MIR100HG may be an oncogenic or tumor suppressor, while the dual role of MIR100HG in cancer may be related to the dual role of TGFβ. Existing studies suggest that TGFβ has antitumor properties in some cancers and tumorigenic effects in others, which deserves to be explored in future studies.

### YAP-hippo signaling pathway

The Hippo signaling pathway is evolutionarily conservative, and YAP is its main effector molecule. Inactivation of the Hippo signaling pathway induces down-regulation of MST1/LATS1 and up-regulation of YAP1, which are the core factors of the pathway. Dysregulation of Hippo signaling has been found in many human tumors and is closely associated with the acquisition of malignant features (82). Su et al. (55) studied the downstream mechanism of MIR100HG and showed that MIR100HG plays an oncogenic role in OS by inactivating the Hippo signaling pathway, as silenced MIR100HG leads to increased MST1, LATS1, LATS2 protein levels and decreased YAP1 protein levels.

### Other signaling pathways

Zapata et al. (56) found that MIR100HG can be considered as a new candidate gene in a risk model of dog osteosarcoma genome scanning, which can function individually or in combination with other genes MYCN, Akt2, MTMR7/9, FGF9, PHLPP1, and BRIP3 also selected in the related PI3K and Rb pathways. Shang et al. (21) further suggested that MIR100HG may be involved in the regulation of LNM in early cervical cancer in various ways. MIR100HG not only participates in the TGF-β pathway, but also participates in gap junction, and most of the mRNAs co-expressed with MIR100HG participate in the gap junction pathway. In multiple PDAC patients, high expression of miR-100 or 125b was correlated with decreased OS and DFS. The two miR-100 or 125b of the MIR100HG host regulate key pathways of PDAC progression, such as TP53, apoptosis, and DNA damage, and also regulate many genes involved in repressing p53 and DNA damage response pathways. These genes are critical for the progression of this common metastatic disease. In the study of gastric cancer, Li et al. (25) showed that MIR100HG is related to muscle tissue and cytoskeletal organization and participates in the interaction of ECM receptors, and these functions have been confirmed to be related to tumor metastasis. MIR100HG is positively associated with vascular smooth muscle contraction, local adhesion, and TGF-β signaling pathways, which is associated with immunosuppression and tumorigenesis, while inversely associated with glutathione metabolism, peroxisome, and glycosphingolipid biosynthesis pathways, mainly involved in reactive oxygen species-induced tumorigenesis.

## MIR100HG and chemoresistance

### Cetuximab

CTX is a monoclonal antibody against EGFR, which binds to the extracellular domain of EGFR and enhances the internalization and degradation of receptors. It is effective for metastatic CRC. When CTX is used as a monotherapy, EGFR monoclonal antibody produces durable responses in 12-17% of patients. When the mAb is combined with chemotherapy, there is a response rate of up to 72%. However, drug resistance frequently emerges with little known about resistance mechanisms (24). Lu et al. (24) established CTX-resistant cell lines of CRC and HNSCC in a three-dimensional medium and found that MIR100HG and its miR-100 and 125b were upregulated in CTX cell lines, and miR-100 and 125b synergistically drive cancer cell resistance to CTX. MiR-100 and 125b can suppress several Wnt inhibitory regulators and upregulate the level of the Wnt signaling pathway, resulting in cell resistance to CTX. This study has important clinical application value and can be used to treat CTX-resistant progressive CRC and HNSCC.

### Sorafenib

Sorafenib (SFB) is a biaryl urea oral multikinase inhibitor, which is a commonly used drug in clinical molecular targeted therapy for liver cancer. Reducing the drug resistance of liver cancer cells has a positive significance for improving the therapeutic efficacy (83). He et al. (83) found that MIR100HG was upregulated and miR-142-5p was downregulated in sorafenib-resistant Huh7/SFB cells compared with Huh7 cells. MIR100HG targets and negatively regulates miR-142-5p expression. Inhibition of MIR100HG expression can improve the inhibition rate of sorafenib on Huh7/SFB cells, promote sorafenib-induced apoptosis and p21 and Bax protein expression in Huh7/SFB cells and inhibit CyclinD1 and Bcl-2 protein expression. Inhibition of miR-142-5p expression could reverse the effects of inhibiting MIR100HG expression on sorafenib-induced Huh7/SFB cell viability, apoptosis, and expression of related proteins p21, Bax, CyclinD1, and Bcl-2. This provides a new idea for improving the drug resistance of liver cancer to SFB and improving the therapeutic efficacy of liver cancer.

### Gemcitabine

Gemcitabine (GEM) is a pyrimidine nucleotide analog, belonging to the antimetabolite class of anticancer drugs. It mainly acts on the DNA S phase, preventing DNA synthesis and leading to cell apoptosis. MiRNAs of the Let-7 family induced EMT reversal in GEM-resistant PDAC cells. MiR-100 and 125b are upregulated in GEM-resistant cells compared with Let-7 family members and promote EMT in PDAC. Targeting non-coding RNAs has been used for anticancer therapy. Inhibiting MIR100HG, miR-100, and 125b can improve PDAC resistance to GEM, which can be regarded as a novel approach to treating PDAC and used as biomarkers for stratified PDAC (44, 45).

### Docetaxel, cisplatin, and S-1

The combination of docetaxel, cisplatin, and S-1 (DCS) is a common chemotherapy regimen for GC. Zhang et al (43) constructed a lncRNA signature including MIR100HG and other 10 lncRNAs, which was associated with the prognosis of GC and can be a more effective prognostic factor for GC patients. Furthermore, this model can well predict chemotherapy drug response and immune infiltration of GC patients. Therefore, the above results have uncovered that MIR100HG combined with other 10 lncRNAs as a new DCS therapy-related lncRNA signature could accurately predict outcomes for gastric cancer patients.

## Summary and prospect

MIR100HG has been proven to be key regulator of human gene expression. Accumulating researchers have reported that MIR100HG is upregulated in many malignant tumors, containing leukemia, head and neck carcinoma, breast carcinoma, pancreatic ductal adenocarcinoma, osteosarcoma, gastric cancer, colorectal cancer, hepatocellular carcinoma and downregulated in a few tumors, including lung cancer, papillary thyroid cancer, and cervical cancer. While MIR100HG is upregulated or downregulated in brain tumor and bladder cancer (**Figure 5**). The regulatory mechanisms of MIR100HG are very complex and involve many steps, including as a promoter of RBPs, as a structural component to form nucleic acid-protein complexes with proteins, as ceRNA, and as a miRNA precursor. MIR100HG mainly regulates the occurrence and development of diseases through the Wnt/β-catenin, ERK/MAPK, TGF-β, YAP-Hippo, and other signaling pathways. MIR100HG played an oncogenic or suppressive role that is involved in various tumor cell biology processes including proliferation, cell cycle, apoptosis, migration and invasion, metastasis, drug resistance, and EMT (**Table 1**). Meanwhile, dysregulated expression of MIR100HG is markedly correlated with poor prognosis and clinicopathological features including tumor size, AJCC stage, TNM stage, LNM, distant metastasis, prognosis, DFS, OS, and chemoresistance (**Table 2**). Therefore, MIR100HG is expected to serve as a promising disease diagnostic and prognostic biomarker or novel treatment target.

While the regulatory mechanisms of MIR100HG in multiple cancers have been investigated, studies on MIR100HG remain in the primary stage, and many key issues need to be further addressed. First and foremost, how to distinguish between effects mediated by MIR100HG and its residing miRNAs. Determining the independent roles that exist for host MIR100HG in different cancers is an open area of investigation. Besides, the reason why MIR100HG can play the opposite role as an inhibitor or promoter in different tumors should be investigated. What’s more, an experimental in-depth study to precisely find the upstream and downstream molecular mechanisms of MIR100HG in different cancers is needed. For example, regulators and targets including mRNA or other miRNAs involved in the aberrant expression of MIR100HG in tumors remain rarely known, and the molecular mechanism of MIR100HG-mediated RNA-binding protein regulation still needs to be clarified in further studies. In addition, there is still a lack of large independent cohort studies for further verification. The physiological role of MIR100HG, the interaction between MIR100HG and the cancer microenvironment, and the function of MIR100HG in immune response, cell metabolism, starvation/autophagy, and neo-vascularization need to be investigated. What’s more, a set of cellular senescence-associated miRNAs (SA-miRNAs) derived from the oncogenic MIR17HG and tumor-inhibiting MIR100HG clusters are effective controllers of complex and coordinated interactions among multiple cellular sub-processes related to cellular senescence. Importantly, it proved the functional significance of these SA-miRNAs to establish an aging phenotype in adult adipose stem cells (84). This raises the crucial issue of whether MIR100HG is involved in the pathogenesis of other diseases related to aging or high-fat metabolism in addition to tumor diseases. Although MIR100HG participates to regulate the progression of the tumor, the biological function of MIR100HG remains largely unknown. The diversification of structure enables RNAs to conduct various functions (85, 86). However, so far, no researchers have focused on the structures of MIR100HG. Thus, it is greatly important to investigate the biological function of MIR100HG in diseases by exploring the structure of MIR100HG.

In summary, we are optimistic that this review will contribute to a better understanding of MIR100HG and its relationship with a variety of cancers and the relevant knowledge can lay a solid foundation for the practical application of MIR100HG in the future.

## Author contributions

YW contributed to the conceptualization, design, formal analysis, and final approval of the submitted version. YW, ZW, and SY collected and analyzed literature. YW, LS, and DL contributed to the manuscript writing. LS contributed to supervision and funding acquisition. All the authors conceived and approved the final manuscript. All authors contributed to the article and approved the submitted version.

## Funding

This work was supported by the National Natural Science Foundation of China (No.82071929).

## Conflict of interest

The authors declare that the research was conducted in the absence of any commercial or financial relationships that could be construed as a potential conflict of interest.

## Publisher’s note

All claims expressed in this article are solely those of the authors and do not necessarily represent those of their affiliated organizations, or those of the publisher, the editors and the reviewers. Any product that may be evaluated in this article, or claim that may be made by its manufacturer, is not guaranteed or endorsed by the publisher.
