# Supplementary material for: LncmiRHG-MIR100HG: A new budding star in cancer
Source: Front Oncol. 2022 Sep 23;12:997532. doi: 10.3389/fonc.2022.997532 (PMC9544809; doi:10.3389/fonc.2022.997532)
Supplement: Supplementary file 1 [file Table_1.docx]

Supplementary Material

**Supplementary Table 1. Abbreviations in Figure 2**

| **Abbreviation** | **Full name** |
| --- | --- |
| TMP | Transcripts per million |
| ESCA | Esophageal carcinoma |
| STAD | Stomach adenocarcinoma |
| COAD | Colon adenocarcinoma |
| READ | Rectum adenocarcinoma |
| LIHC | Liver hepatocellular carcinoma |
| CHOL | Cholangiocarcinoma |
| PAAD | Pancreatic adenocarcinoma |
| LUAD | Lung adenocarcinoma |
| LUSC | Lung squamous cell carcinoma |
| BLCA | Bladder urothelial carcinoma |
| KICH | Kidney Chromophobe |
| KIRC | Kidney renal clear cell carcinoma |
| KIRP | Kidney renal papillary cell carcinoma |
| BRCA | Breast invasive carcinoma |
| CESC | Cervical squamous cell carcinoma and endocervical adenocarcinoma |
| OV | Ovarian serous cystadenocarcinoma |
| UCEC | Uterine corpus endometrial carcinoma |
| UCS | Uterine carcinosarcoma |
| PRAD | Prostate adenocarcinoma |
| TGCT | Testicular germ cell tumors |
| LAML | Acute myeloid leukemia |
| DLBC | Diffuse large B-cell lymphoma |
| GBM | Glioblastoma multiforme |
| LGG | Brain lower grade glioma |
| SARC | Sarcoma |
| PCPG | Pheochromocytoma and paraganglioma |
| HNSC | Head and neck squamous cell carcinoma |
| SKCM | Skin cutaneous melanoma |
| THCA | Thyroid carcinoma |
| THYM | Thymoma |
| ACC | Adrenocortical carcinoma |

**Supplementary Table 2. Abbreviations in Figure 3**

| **Abbreviation** | **Full name** |
| --- | --- |
| HR | Hazards ratio |
| ACC | Adrenocortical carcinoma |
| BLCA | Bladder urothelial carcinoma |
| BRCA | Breast invasive carcinoma |
| CESC | Cervical squamous cell carcinoma and endocervical adenocarcinoma |
| CHOL | Cholangiocarcinoma |
| COAD | Colon adenocarcinoma |
| DLBC | Diffuse large B-cell lymphoma |
| ESCA | Esophageal carcinoma |
| GBM | Glioblastoma multiforme |
| HNSC | Head and neck squamous cell carcinoma |
| KICH | Kidney Chromophobe |
| KIRC | Kidney renal clear cell carcinoma |
| KIRP | Kidney renal papillary cell carcinoma |
| LAML | Acute myeloid leukemia |
| LGG | Brain lower grade glioma |
| LIHC | Liver hepatocellular carcinoma |
| LUAD | Lung adenocarcinoma |
| LUSC | Lung squamous cell carcinoma |
| MESO | Mesothelioma |
| OV | Ovarian serous cystadenocarcinoma |
| PAAD | Pancreatic adenocarcinoma |
| PCPG | Pheochromocytoma and paraganglioma |
| PRAD | Prostate adenocarcinoma |
| READ | Rectum adenocarcinoma |
| SARC | Sarcoma |
| SKCM | Skin cutaneous melanoma |
| STAD | Stomach adenocarcinoma |
| TGCT | Testicular germ cell tumors |
| THCA | Thyroid carcinoma |
| THYM | Thymoma |
| UCEC | Uterine corpus endometrial carcinoma |
| UCS | Uterine carcinosarcoma |
| UVM | Uveal melanoma |
